# Differential alterations of the concentrations of endocannabinoids and related lipids in the subcutaneous adipose tissue of obese diabetic patients

**DOI:** 10.1186/1476-511X-9-43

**Published:** 2010-04-28

**Authors:** Giovanni Annuzzi, Fabiana Piscitelli, Lucrezia Di Marino, Lidia Patti, Rosalba Giacco, Giuseppina Costabile, Lutgarda Bozzetto, Gabriele Riccardi, Roberta Verde, Stefania Petrosino, Angela A Rivellese, Vincenzo Di Marzo

**Affiliations:** 1 Department of Clinical and Experimental Medicine, Federico II University, Naples, Italy; 2 Endocannabinoid Research Group, Institute of Biomolecular Chemistry, CNR, Pozzuoli Naples, Italy; 3 National Research Council, Avellino, Italy

## Abstract

**Background:**

The endocannabinoids, anandamide and 2-AG, are produced by adipocytes, where they stimulate lipogenesis via cannabinoid CB_1 _receptors and are under the negative control of leptin and insulin. Endocannabinoid levels are elevated in the blood of obese individuals and nonobese type 2 diabetes patients. To date, no study has evaluated endocannabinoid levels in subcutaneous adipose tissue (SAT) of subjects with both obesity and type 2 diabetes (OBT2D), characterised by similar adiposity and whole body insulin resistance and lower plasma leptin levels as compared to non-diabetic obese subjects (OB).

**Design and Methods:**

The levels of anandamide and 2-AG, and of the anandamide-related PPARα ligands, oleoylethanolamide (OEA) and palmitoylethanolamide (PEA), in the SAT obtained by abdominal needle biopsy in 10 OBT2D, 11 OB, and 8 non-diabetic normal-weight (NW) subjects, were measured by liquid chromatography-mass spectrometry. All subjects underwent a hyperinsulinaemic euglycaemic clamp.

**Results:**

As compared to NW, anandamide, OEA and PEA levels in the SAT were 2-4.4-fold elevated (p < 0.05), and 2-AG levels 2.3-fold reduced (p < .05), in OBT2D but not in OB subjects. Anandamide, OEA and PEA correlated positively (p < .05) with SAT leptin mRNA and free fatty acid during hyperinsulinaemic clamp, and negatively with SAT LPL activity and plasma HDL-cholesterol, which were all specifically altered in OBT2D subjects.

**Conclusions:**

The observed alterations emphasize, for the first time in humans, the potential different role and regulation of adipose tissue anandamide (and its congeners) and 2-AG in obesity and type 2 diabetes.

## Background

The endocannabinoid system (ECS), composed of G-protein-coupled cannabinoid receptors of type 1 and 2 (CB_1 _and CB_2_), of endogenous ligands for such receptors, the endocannabinoids arachidonoylethanolamide (anandamide, AEA) and 2-arachidonoylglycerol (2-AG), and of enzymes catalysing endocannabinoid biosynthesis and degradation, is a key player in the control of metabolism at both central and peripheral levels [1]. In the hypothalamus, endocannabinoids acting at CB_1 _receptors modulate the circuitries involved in food intake and are under the negative control of leptin [2]. In the white adipose tissue (WAT), the ECS stimulates lipogenesis and inhibits lipolysis via several mechanisms, and is under the negative control of either leptin or PPARγ [1,3-5]. PPARγ agonists, such as the glitazones, used for the treatment of type 2 diabetes (T2D), reduce either adipocyte 2-AG concentrations or CB_1 _receptor expression levels, or both [3-5]. Insulin also acts as a negative modulator of endocannabinoid levels in both human and murine WAT [3,6,7]. Plasma endocannabinoid levels, which likely reflect to a large extent the "spill-over" of these lipid mediators from peripheral organs, are decreased postprandially or following oral glucose load and euglycaemic hyperinsulinaemic clamp in lean [3,8], but not in insulin resistant obese subjects [8], who, like T2D patients, show higher levels of endocannabinoids also when fasting [3-7,9].

In abdominally obese individuals, blood 2-AG, but not AEA, concentrations correlate directly with the amount of visceral adipose tissue (VAT) and blood triacylglycerols (TGs), and inversely with HDL-cholesterol levels [10,11], and such correlations are also observed between the changes in blood 2-AG levels and those in TG and HDL-cholesterol levels induced by a lifestyle intervention leading to a strong reduction of waist circumference and VAT [12]. In experimental models of obesity, higher levels of endocannabinoids are found also in the visceral (e.g. epididymal) *vs. *subcutaneous adipose depots [3,13,14]. This dysregulation of endocannabinoid tone in the WAT is probably due, at least in part, to dysfunctional expression of endocannabinoid metabolising enzymes. In fact, a lower expression of the fatty acid amide hydrolase (FAAH), which can metabolise both AEA and 2-AG, as well as other congeners of AEA (see below), or of the monoacylglycerol lipase (MAGL), which is more specific for 2-AG [15], has been reported in the VAT of obese individuals [10,16], possibly accounting for the observed higher VAT levels of 2-AG [3]. As opposed to obese rodents [13], reduced levels of FAAH expression have been reported for obese subjects also in the subcutaneous adipose tissue (SAT) [9,16,17], where, instead, there seems to be no elevation of endocannabinoid levels [3]. However, no such studies have been performed to date in the WAT of obese patients with type 2 diabetes, who can be characterised by similar whole body insulin resistance, but lower plasma leptin levels, as compared to matched non diabetic obese subjects [18,19].

The SAT plays an important role as a buffer against ectopic lipid accumulation and the subsequent increased inflammatory profile typical of abdominally obese patients [20]. Therefore, in view of the prolipogenic role of the ECS in the WAT, a higher endocannabinoid tone in SAT might be regarded as protective towards the metabolic consequences of obesity, whereas a reduced tone might contribute to these consequences. For this reason, and also to acquire some unprecedented information on endocannabinoid tone in the WAT of subjects with T2D, we have compared here the levels of AEA and 2-AG in the SAT of age- and gender-matched T2D obese (OBT2D), obese only (OB), and normal weight (NW) volunteers. Furthermore, since two cannabinoid receptor-inactive and metabolically-related AEA congeners, oleoylethanolamide (OEA) and palmitoylethanolamide (PEA), which are also degraded by FAAH, are emerging as potent endogenous ligands of peroxisome proliferator-activated receptor α (PPARα), a well established target for the therapy of dyslipidemia [21], and were recently found to be dysregulated in obese Zucker rats [22], we have also measured the SAT levels of these two lipid mediators.

## Methods and subjects

### Subjects

Ten patients with type 2 diabetes mellitus and obesity (OBT2D), 11 patients with only obesity (OB), and 8 normal-weight control subjects (NW) participated in the study. Their baseline characteristics are shown in Table 1. All subjects had normal fasting plasma concentration of both triglyceride (<1.7 mmol/l) and cholesterol (<5.5 mmol/l). The subjects had no history or symptoms of any known disease, apart from diabetes, nor were they vegetarians or engaged in intensive physical activity. They were not taking any hypolipidaemic drug. Diabetic patients were in stable glycaemic control on diet alone (HbA1c = 6.5 ± 0.5%). The study protocol was approved by the Federico II University Ethics Committee and informed consent by the participants was obtained.

**Table 1:** Clinical characteristics and metabolic measurements in plasma and adipose tissue of the subjects participating in the study.

	Diabetes + obesity	Obesity	Control
Males (n)	10	11	8

Age (years)	47 [8]	47 [20]	38 [9]

BMI (kg/m^2^)	33.4 [2.4] **	34.5 [2.7] **	23.8 [1.2]

Waist circumference (cm)	111.4 [7.7] **	113.1 [6.7] **	84.9 [4.4]

Plasma cholesterol (mg/dl)	171.8 [25.9]	194.1 [29.9]	166.7 [24.2]

Plasma triglyceride (mg/dl)	111.2 [54.7]	99.8 [34.7]	72.4 [28.2]

LDL cholesterol (mg/dl)	102.1 [19.6]	114.9 [26.4]	96.8 [14.6]

HDL cholesterol (mg/dl)	34.5 [5.8] *§	44.3 [10.3]	47.7 [10.6]

Plasma glucose (mg/dl)	132.7 [37.2] *§	87.9 [13.9]	87.2 [9.9]

Plasma insulin (mU/l)	14.3 [6.4]	15.6 [4.2]	6.4 [1.5]

Plasma leptin (ng/ml)	9.4 [4.2]§	24.9 [9.6]**	5.4 [2.3]

Adipose tissue leptin mRNA (AU)	2.62 [1.90] *	1.73 [1.82]	0.77 [0.38]

Adipose tissue LPL activity (nmol fatty acids/g tissue)	105 [45] *§	231 [128]	251 [119]

Insulin sensitivity (M-value) (mg/kg/min)	4.55 [1.41] **	4.63 [1.48] **	8.13 [2.32]

FFA during hyperinsulinaemic clamp (μmol/l)	84.0 [50.8] *	73.0 [33.7] *	36.2 [7.4]

Data are expressed as M [SD]. ANOVA and LSD (Kruskal-Wallis for plasma triglyceride, LPL activity, and leptin RNA) * p < 0.05, ** p < 0.001 vs Control, §p < 0.05 vs Obesity. AU = arbitrary units, LPL = lipoprotein lipase.

### Experimental procedures

In the morning, after a 12-14 hr fast, following an evening meal based on the usual diet, anthropometric measurements, according to standardized procedures, and blood samples were drawn. Thereafter, a needle biopsy of the abdominal subcutaneous adipose tissue was taken under local anaesthesia with lidocaine, and a hyperinsulinaemic euglycaemic clamp was performed (120 min insulin infusion 1.5 mU·kg body weight^-1^·min^-1^) [18]. Advice was given to have the meal at the proper time, and to avoid alcohol intake. Compliance was assessed by a 3-day food diary filled in by the participants. The subjects were also requested to avoid moderate/strenuous physical activity on the 3 days before the experiment.

### Extraction and quantification of endocannabinoids and related lipids

Frozen SAT tissue samples were homogenized in chloroform/methanol/TRIS-HCl 50 mM pH 7.4 (2:1:1, v/v) containing 10 pmol of [^2^H]_8_-AEA, [^2^H]_4_-PEA and [^2^H]_4_-OEA, and 50 pmol of [^2^H]_5_-2-AG as internal deuterated standards (purchased from Cayman Chemicals, Ann Arbor, MI). The extract was purified by means of silica gel mini-columns, and the eluted fraction containing AEA and 2-AG analysed by means of liquid chromatography-atmospheric pressure-mass spectrometry (LC-APCI-MS) conducted as described previously [23]. Analyses were carried out in the selected ion-monitoring mode using *m/z *values of 356 and 348 (molecular ions +1 for deuterated and undeuterated AEA), 304 and 300 (molecular ions +1 for deuterated and undeuterated PEA), 330 and 326 (molecular ions +1 for deuterated and undeuterated OEA), and 384.35 and 379.35 (molecular ions +1 for deuterated and undeuterated 2-AG). AEA, OEA, PEA and 2-AG concentrations were calculated by isotope dilution and are expressed as pmol per g of wet tissue weight. The concentrations of 2-AG were obtained by adding up to the amounts of the 2-isomer also those of the 1(3)-isomer, which mostly originates from the isomerization of the former during work-up.

### Other measurements

Plasma cholesterol and triglyceride concentrations were assayed by enzymatic colorimetric methods (Roche Diagnostics, Milan, Italy). Plasma leptin concentrations were assayed using a competitive ELISA (BioVendor Laboratory Medicine, Brno, Czech Republic). Plasma insulin concentrations were measured by ELISA (Technogenetics, Milan, Italy).

LPL heparin-releasable activity in adipose tissue was determined as previously described [24]. The expression of leptin mRNA was evaluated by RT-PCR [25]. The specific primers to amplify leptin (Acc.U43653) were sense: 5'-ATG CAT TGG GGA ACC CTG TGC GG-3'and antisense: 5'-AGG TCC AGC TGC CAC AGC ATG TC-3' (487 bp). Results are expressed as the ratio between the leptin gene and GAPDH in each sample analyzed.

### Statistical analyses

Data are expressed as mean ± standard deviation (M ± SD), unless otherwise stated. Differences between the three groups (OBT2D, OB and NW) were evaluated by ANOVA and by post hoc test between groups (LSD). The relationship between endocannabinoid levels and metabolic measurements was assessed by Pearson correlation. Variables not normally distributed were analyzed by a nonparametric test (Kruskal-Wallis ANOVA). Two-tailed tests were used and a p < 0.05 was considered statistically significant. Statistical analysis was performed according to standard methods using the Statistical Package for Social Sciences software (SPSS/PC, SPSS, Inc., Chicago, IL).

## Results

### Anthropometrics and metabolic measurements

Obese subjects with and without diabetes had similarly high BMI and abdominal circumferences compared with NW controls (Table 1). As expected, blood glucose levels were significantly higher in OBT2D group compared to the other two groups. Fasting plasma insulin levels were significantly higher in both groups of obese with and without diabetes compared to NW.

Insulin sensitivity measured by euglycaemic hyperinsulinaemic clamp and expressed as M-value was similarly lower in the two obese groups compared to NW controls. Insulin antilipolytic activity, expressed by plasma FFA level inhibition during the hyperinsulinaemic clamp, was impaired in the OBT2D subjects compared to the other two groups.

Fasting plasma leptin levels where highest, intermediate and lowest in OB, OBT2D and NW subjects, respectively, while leptin mRNA in adipose tissue tended to be higher in OBT2D than in NW. Heparin-releasable LPL activity, expressed per gram of adipose tissue, was significantly lower in the OBT2D subjects compared to the other two groups.

### Adipose tissue endocannabinoid levels

**Figure 1:**
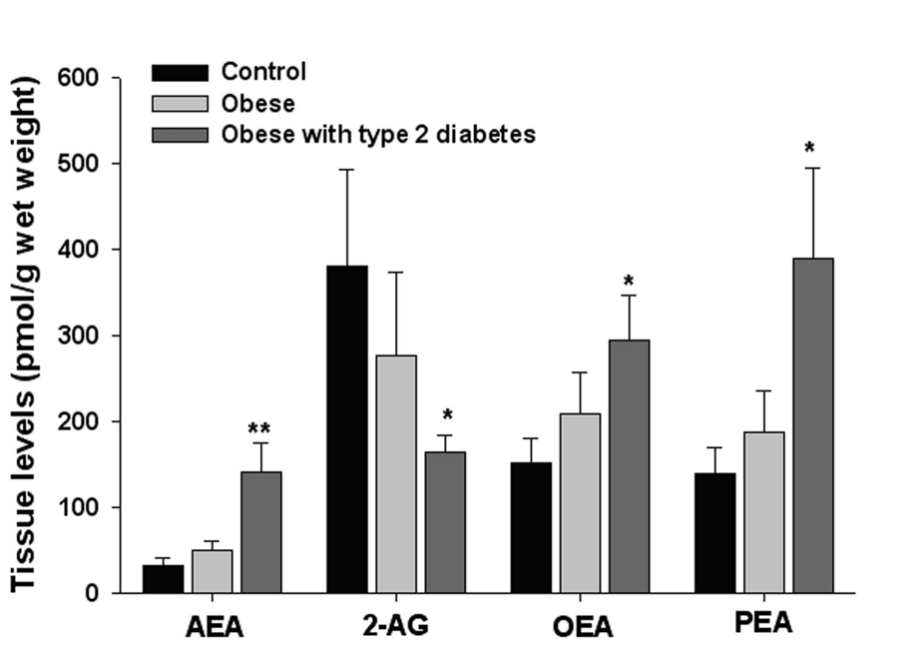
**Differential alterations of endocannabinoid, oleoylethanolamide and palmitoylethanolamide levels in the subcutaneous adipose tissue of normoweight, obese and type 2 diabetes obese subjects**. Endocannabinoid levels (in pmol/g tissue, means ± SE) were measured in the subcutaneous adipose tissue in individuals with obesity and diabetes (N = 10), only obesity (N = 11), and normal weight controls (N = 8). AEA = anandamide (arachidonoylethanolamide); 2-AG = 2-arachidonoylglycerol; OEA = oleoylethanolamide; PEA = palmitoylethanolamide. ANOVA and LSD (Kruskal-Wallis for 2-AG) *p < 0.05, **p < 0.001 *vs. *Control and Obese.

As shown in Fig. 1, the SAT concentrations of AEA, OEA and PEA were significantly higher in OBT2D than in either OB or NW subjects. By contrast, 2-AG concentrations were significantly lower in OBT2D than in either OB or NW subjects. No significant differences were observed between the concentrations of these four compounds in the SAT of OB and NW subjects.

In the three groups combined, OEA and PEA concentrations in the SAT correlated positively with each other and with AEA, whereas no correlation was found between AEA, OEA or PEA levels and 2-AG (Table 2). AEA, OEA and PEA levels were positively correlated with plasma FFA during hyperinsulinaemic clamp and SAT leptin RNA; and negatively correlated with HDL cholesterol and adipose tissue LPL activity. Adipose tissue 2-AG levels were positively correlated with M-value.

**Table 2:** Relationship between endocannabinoid levels in adipose tissue (expressed as pmol/g tissue) and metabolic measurements in all subjects participating in the study (Pearson correlation coefficients).

	AEA	2-AG	OEA	PEA
AEA		-.10	.76***	.87***

2-AG			.32	.09

OEA				.87***

Plasma triglycerides	.27	-.11	.25	.26

HDL cholesterol	-.53**	.10	-.48**	-.47**

Plasma leptin	-.19	-.16	-.09	-.12

Leptin mRNA in SAT	.44*	.18	.70***	.62***

LPL activity in SAT	-.53**	.30	-.50**	-.48**

Insulin sensitivity (M value)	-.32	.50**	-.17	-.19

FFA during hyperinsulinaemic clamp	.47*	-.05	.55**	.57***

* p < 0.05, ** p < 0.01, *** p < 0.001

## Discussion

In the present study we have measured for the first time the concentrations of two cannabinoid receptor agonists, AEA and 2-AG, and two AEA-related PPAR-α activators, OEA and PEA, in the SAT of obese individuals, with or without T2D, and in NW subjects. Although several other congeners of AEA (known as *N*-acylethanolamines) and 2-AG (the monoacylglycerols) occur in tissues, we quantified here AEA, OEA, PEA and 2-AG because these are the most studied members of their families of lipids, the molecular target of which are also more relevant to the pathophysiology of energy metabolism.

### Possible significance of the observed alterations of the concentrations of endocannabinoids and related lipids in the SAT of OBT2D patients

In the only study published to date in which endocannabinoid levels have been measured in the SAT of obese patients [3], no differences were observed with the corresponding values in matched NW volunteers, and we have confirmed here this finding. However, we have also found that, in the presence of T2D, obese patients do exhibit differential alterations of the SAT concentrations of the four measured lipid mediators. In fact, whilst the SAT levels of the endocannabinoid AEA and of the PPARα ligands OEA and PEA were significantly higher in OBT2D, those of the other, more abundant, endocannabinoid, 2-AG, were lower. OEA and PEA activate PPARα receptors with high and intermediate potency, respectively, but are inactive at cannabinoid receptors [26]. Therefore, the SAT of OBT2D subjects might be characterized by a relatively decreased activity of CB_1 _receptors, and strongly increased activity of PPARα receptors, as compared to that of OB and NW subjects. Given the pro- and anti-lipogenic effects of CB_1 _and PPARα receptor activation, respectively [3,27], it is tempting to hypothesize that this alteration in the activity of the two receptors might be partly responsible for the decreased subcutaneous *vs. *visceral fat observed in OBT2D patients [28].

### Possible explanations for the observed differential alterations of endocannabinoid levels in the SAT of OBT2D patients

It is not unusual that AEA and 2-AG tissue concentrations change in different, or even opposing, ways in physiological and pathological conditions, and in both laboratory animals and humans [29]. This probably reflects the fact that the two compounds might also play different roles, particularly as AEA is known to interact also with non-cannabinoid receptors, including transient receptor potential vanilloid type-1 channels [30] and, at higher concentrations, PPAR-γ [31], whereas 2-AG is more selective for cannabinoid receptors. Furthermore, AEA, OEA, and PEA share similar biosynthetic and inactivation pathways and enzymes (including FAAH) [32], which might explain why the levels of these three compounds were found here to be positively correlated with each other and were altered in the same way in the SAT of OBT2D subjects. The formation and degradation of 2-AG is, instead, catalysed by other enzymes [32]. Interestingly, by acting as competing substrates for FAAH, OEA and PEA might spare AEA and 2-AG from degradation and hence affect endocannabinoid tone. Finally, the different availability of precursor fatty acid pools can differentially affect the levels of *N*-acylethanolamines and monoacylglycerols, including AEA and 2-AG [33], and this can also explain differential changes in levels of the two endocannabinoids in the three populations studied. Indeed, it was previously described that there are alterations in arachidonic acid metabolism in obesity [34], and since arachidonic acid is a precursor to inflammatory eicosanoids, and inflammation is generally increased in obesity and diabetes, this could also account for an altered availability of arachidonic acid for endocannabinoid production.

### Possible meaning of the observed correlations between SAT levels of endocannabinoids **or **related lipids and various biochemical parameters

Previous observations show that: 1) in nonobese, but not in obese, individuals, insulin negatively controls AEA more significantly than 2-AG blood levels [8], possibly by stimulating the expression of FAAH [6], which is more specific for AEA than 2-AG; and 2) circulating leptin levels are negatively associated with blood AEA, but not 2-AG, levels in lean, but not in obese, women with eating disorders [35], although leptin reduces both AEA and 2-AG levels in mouse adipocytes [3]. In the present study OBT2D subjects had lower circulating leptin than OB subjects. This indicates that the increase in SAT AEA levels (and, possibly, also OEA and PEA), observed in OBT2D subjects was more related to the lower leptin levels than to the level of whole body insulin resistance, which was instead similar between the two obese groups. Interestingly, however, the concentrations of AEA, OEA and PEA, did not correlate negatively with plasma leptin levels, nor with M values, but did correlate positively with SAT leptin mRNA levels. This might suggest a possible negative feed-back loop of these lipid mediators on their own levels, exerted by up-regulating the expression and production of one of their negative effectors. Indeed, activation of CB_1 _receptors was shown to stimulate insulin release from both rat and human β-cells [3,36], thus indicating that such negative feed-back regulations are not unusual for the ECS. With regard to 2-AG, here positively correlated with M-values, the reduced whole body insulin sensitivity cannot explain the reduction of its levels in the SAT of OBT2D subjects, since these subjects exhibited M-values similar to those of OB subjects. This does not exclude other effects of reduced insulin sensitivity at any other level in the SAT of OBT2D subjects.

OBT2D subjects had lower adipose tissue LPL activity than the matched OB or NW subjects. It has been shown that LPL in adipocytes is stimulated by the activation of CB_1 _receptors [37]. Therefore, since SAT LPL activity here correlated negatively with SAT AEA (and OEA or PEA) levels, while 2-AG levels showed an opposite tendency, it is possible to hypothesize, again, that increased AEA might not result in increased SAT CB_1 _activity, whereas decreased levels of 2-AG in the SAT might be partly responsible, via the corresponding reduction of CB_1 _activity, for the decreased LPL activity observed in this adipose depot. This hypothesis would be in agreement also with the very recent finding of reduced CB_1 _receptor mRNA expression in the SAT of both obese [38] and overweight dysmetabolic subjects [39], and with mounting evidence that CB_1 _receptor tone is reduced in the SAT of obese animals [13,14].

The SAT concentrations of AEA, but not 2-AG, correlated with decreased HDL cholesterol, which is the opposite of what previously reported for the plasma levels of 2-AG, which, unlike plasma AEA levels, are correlated with decreased HDL cholesterol [10-12]. The SAT concentrations of AEA also correlated with a biomarker that is usually linked with increased lipogenesis in the liver, i.e. plasma FFA during hyperinsulinaemic clamp. Therefore, it cannot be excluded that some of the metabolic dysfunctions observed in OBT2D subjects, which might be caused by overactivation of CB_1 _receptors, are also due to elevated AEA concentrations in the SAT. This, in turn, might lead to the release of this endocannabinoid in the circulation and to its action on other target tissues (e.g. the liver), in which CB_1 _receptor expression/function is not reduced. In this study we did not measure the levels of AEA and 2-AG in other tissues or in the plasma. A previous study carried out in post-menopausal obese women showed that increased plasma endocannabinoids levels correlate with decreased FAAH mRNA expression in the SAT [9], which, in turn, should result in increased SAT endocannabinoid levels. However, in viscerally obese men, only increased plasma 2-AG levels correlated with decreased FAAH mRNA expression in the SAT [10]. Furthermore, plasma 2-AG, but not AEA, levels are significantly increased in the plasma of OBT2D *vs. *OB or T2D male subjects (V. Di Marzo and L. Van Gaal, unpublished observations). Therefore, in obese subjects such as those investigated in the present study, little correlation is likely to exist between circulating and SAT endocannabinoid levels. In this respect, the decrease in 2-AG concentrations found here in the SAT of OBT2D subjects is in contrast with the increase in circulating 2-AG levels, observed previously in overweight T2D patients [3]. Also the aforementioned positive correlation between SAT 2-AG levels and M-values is in contrast with what observed with plasma 2-AG levels in obese subjects [8,10]. In fact, SAT and plasma 2-AG levels seem to predict in opposing ways insulin resistance and T2D. Likewise, although both SAT (this study) and blood [3] AEA concentrations are elevated in T2D patients, the amounts of FFA during hyperinsulinaemic clamp correlated positively with SAT AEA (this study) and negatively with blood AEA [10].

## Conclusions

The concentrations of endogenous lipid mediators that exert their biological actions by activating either CB_1 _or PPARα receptors are altered in the SAT of OBT2D, but not of simply obese, individuals. Based on our current knowledge of the role of the ECS in the WAT [1,4,5], such alterations might contribute to less fat accumulation in the SAT relative to VAT, and to some metabolic alterations that, along with impaired insulin release and sensitivity, are typical of obese patients with T2D [40,41].

## List of abbreviations

2-AG: 2-arachidonoylglycerol; AEA: anandamide; BMI: body mass index; CB_1_: cannabinoid receptor type 1; EC: endocannabinoid; ECS: endocannabinoid system; FAAH; fatty acid amide hydrolase; FFA: free fatty acid; HbA1c: glycated haemoglobin; HDL: high density lipoprotein; LPL: lipoprotein lipase; MAGL: monoacylglycerol lipase; NW: normoweight; OB: obese; OBT2D: obese with type 2 diabetes; OEA: oleoylethanolamide; PEA: palmitoylethanolamide; PPAR: peroxisome proliferator-activated receptor; SAT: subcutaneous adipose tissue; T2D; type 2 diabetes; TG: triacylglycerol; VAT: visceral adipose tissue; WAT: white adipose tissue.

## Competing interests

The authors declare that they have no competing interests.

## Authors' contributions

GA, AAR, GR and VD designed the study; FP, LD, LP, RG, GC, LB, RV and SP performed the experiments; GA, FP and VD wrote the paper. All authors read and approved the final manuscript.
